# *Leptospira* biofilms: implications for survival, transmission, and disease management

**DOI:** 10.1128/aem.01914-24

**Published:** 2025-01-10

**Authors:** Carla Silva Dias, Melissa Hanzen Pinna

**Affiliations:** 1 Postgraduate Program in Animal Science in the Tropics - Federal University of Bahia 28111 , Salvador, Bahia, Brazil; Centers for Disease Control and Prevention, Atlanta, Georgia, USA

**Keywords:** biofilm, *Leptospira*, leptospirosis, antimicrobial resistance, chronic infection

## Abstract

Leptospirosis is a zoonotic disease caused by *Leptospira* bacteria, affecting humans and a broad range of wild and domestic animals in diverse epidemiological settings (rural, urban, and wild). The disease’s pathogenesis and epidemiology are complex networks not fully elucidated. Epidemiology reflects the One Health integrated approach of environment–animal–human interaction, causing severe illness in humans and animals, with consequent public health burdens. Saprophytic and pathogenic leptospires have been shown to form biofilms *in vivo*, *in vitro,* and in environmental samples. Biofilms are characterized by a polymeric matrix that confers protection against hostile environments (both inside and outside of the host), favoring bacterial survival and dissemination. Despite its significance, the role of this bacterial growth mode in leptospiral survival, transmission, and decreased antibiotic susceptibility remains poorly understood and underexplored. Even so, the literature indicates that biofilms might be correlated with lower antimicrobial susceptibility and chronicity in leptospirosis. In this minireview, we discuss the aspects of biofilm formation by *Leptospira* and their significance for epidemiology and therapeutic management. Understanding the current scenario provides insight into the future prospects for biofilm diagnosis, prevention, and treatment of leptospirosis.

## INTRODUCTION

Leptospirosis is a widespread zoonosis, impacting diverse populations and ecosystems, with over 1.03 million human cases estimated globally (1). Leptospirosis is caused by environmentally ubiquitous bacteria of the genus *Leptospira* and affects humans and various animal species, which can be either incidental hosts or chronic carriers. The disease plays a significant role at the human–animal–environment interface, leading to important consequences for One Health (2, 3).

Leptospirosis is multisystemic, but despite progress in studying *Leptospira*–host interactions, the various mechanisms of infection and pathogenesis remain incompletely understood (4). One mechanism involved in renal colonization is the formation of bacterial biofilms, which favors immune evasion, chronic colonization, and antibiotic resistance (5–7).

Biofilms are communities of bacteria organized as aggregates protected by an extracellular polymeric substance (EPS) (8). Biofilm growth involves a complex process that helps bacteria persist in the environment and hosts, making infections harder to treat and prevent. Therefore, understanding how *Leptospira* form biofilms and identifying the research gaps are key to developing more assertive strategies for biofilm detection, prevention, and control of leptospirosis. This is especially crucial in endemic areas where leptospirosis poses a significant public health burden.

## *LEPTOSPIRA* SPP.

The causative agents of leptospirosis are aerobic bacteria of the order Spirochaetales and genus *Leptospira*. These Gram-negative organisms, characterized by their spiral shape, possess lipopolysaccharides in their outer membrane and feature two periplasmic flagella that enable motility (9). Each flagellum has a hook-shaped end and measures 0.1 µm in diameter and between 6 and 20 µm in length. Currently, over 300 distinct serovars are recognized based on antigenic determinants of the lipopolysaccharide components (3). Genotypically, there are 69 species divided into pathogenic, intermediate, and saprophytic (10–13).

Pathogenic species multiply in the renal tubules of host animals (14) and are excreted into the environment through urine. Humans and susceptible animals can be directly contaminated through contact with infected urine or indirectly through environmental exposure to contaminated soil or water (15). *Leptospira* are fastidious bacteria that require specific conditions for growth, isolation, and *in vitro* cultivation (16). While *Leptospira* can survive in the environment (17), more studies are needed to link the environmental survival of pathogenic species to infections in humans and animals (18).

The determinants for the epidemiological patterns of leptospirosis include a lack of sanitary and health infrastructure, inadequate urbanization, heavy rainfall and flooding, and social-cultural influences (19). Geographically, countries in Southeast Asia, South America, Sub-Saharan Africa, and Oceania report high endemicity (1, 20). Each year, the burden of leptospirosis in humans was estimated at over one million cases, resulting in more than 60,000 fatalities globally (1). Particularly during outbreaks, case fatality for severe Weil’s disease and pulmonary hemorrhage can exceed 10% mortality, reaching over 50% in untreated cases (21). Mild cases are often misdiagnosed because they resemble other febrile illnesses such as dengue and malaria, leading to a significant number of unrecognizable cases (22).

*Leptospira* infects a wide variety of animal reservoir hosts (23). In dogs, severe disease resembles that of human severe cases. For asymptomatic infections, the seroprevalence has been estimated as 19.5% and 9.7% for dogs and cats, respectively (24). The disease also affects livestock globally, with reported high prevalence in Europe and Latin America (25–27), where it is recognized as the major cause of abortions, stillbirths, and infertility, leading to substantial economic losses (2). Currently, varied diagnostic techniques are employed across different epidemiological studies, which complicates the determination of an accurate disease burden. Leptospirosis surveillance should be enhanced based on integrated data on epidemiological trends for effective prevention and control in both humans and animals.

## BIOFILMS

Bacteria were previously classified into a single growth form—planktonic—which is characterized by individual, free-living cells. However, in 1978, the theory of biofilm formation was postulated through the observation of aquatic ecosystems where most bacteria appeared to grow adhered to surfaces and contained in a matrix (28). Advances in microscopy and molecular methods revealed variability in biofilm structure and composition, leading to a more generic definition: microbial cells immobilized in a self-produced extracellular matrix, forming a functional, dynamic ecosystem that interacts with the surrounding environment (29).

Biofilm development into a mature phenotype is a multi-stage process influenced by factors like the microorganisms present, attachment surface, nutrient supply, pH, temperature, and gene expression (30). Given these variables and the impact of different environments on biofilms, biofilm formation is thought to involve three key events: aggregation, growth, and dispersion (8). Briefly, bacterial aggregation is followed by the production of an exopolymeric matrix, which seizes the initial colonizing microorganisms and acts as a receptor for the adhesion of new cells (31). Cell accumulation culminates in the formation of microcolonies, where lifestyle and cellular metabolic activities are coordinated through the process of quorum sensing (QS) (32).

Quorum sensing is a cell-to-cell communication system where signaling molecules coordinate bacterial behaviors, such as biofilm formation and the expression of pathogenicity mechanisms (32). In *Leptospira*, it has been proposed that QS plays a role in regulating biofilm formation in both environmental settings and animal hosts (6). Understanding how these molecules respond to and influence the behavior of leptospires may provide new therapeutic targets aimed at biofilm disruption.

During maturation, extracellular polymeric substance (EPS) production increases. This highly hydrated matrix, composed of polysaccharides, lipids, nucleic acid, and inorganic substances, supports the maintenance of the biofilm’s structure and protects its composing organisms (33). The main component is extracellular DNA (eDNA), mainly responsible for biofilm architecture, gene transfer, DNA repair, and QS (32). Biofilm maturation crowns in a complex architecture characterized by water channels that allow the circulation of nutrients, water, oxygen, and inorganic salts while also enabling the removal of metabolic waste (34). At the last stage, bacterial cells separate from the main biofilm mass through physical (erosion) and chemical (exchange of substrates and nutrients) mechanisms (32). Scattering of bacterial cells is valuable to promote the colonization of new niches, a relevant survival strategy.

While planktonic forms contribute to proliferation and dissemination, sessile bacterial communities enable persistence in the host and the environment (35). The structure of biofilms provides bacterial protection against hostile surroundings (inside or outside hosts), favoring bacterial survival and dissemination (36). As biofilms are found in a variety of natural environments (marine, freshwater, and effluents) and clinical conditions (infections in humans and animals) (36), they are closely connected to the concept of One Health. This unifying approach encompasses the human–animal–environment interface, recognizing that their health is interdependent. Moreover, breaking down the barriers that separate humans and veterinary medicine from environmental sciences and socioeconomic aspects conforms to the transmission cycle of leptospirosis (27). As with many zoonotic diseases, *Leptospira* navigates these scenarios in an integrated way, being biofilm-producers and disseminated in this shared environment between animals and humans (37).

The association of biofilms with chronicity and, therefore, the maintenance of sources of infection, highlight their relevance within the One Health framework. In leptospirosis, the integration of environmental, human, and animal health is contemplated in its epidemiology (22). Chronically infected animals intermittently shed pathogenic *Leptospira* into the environment (urban, rural, and wildlife), introducing a persistent source of contamination and facilitating interspecies circulation. Notably, biofilm disruption resulted in the maintenance of virulence from detached cells (38). This cycle is especially significant in areas with poor sanitation and high disease rates, where humans and animals are in close contact (1). Moreover, biofilms can survive under diverse stressful environmental conditions, becoming potential perpetrators to a wide range of human and animal hosts (38). It is well established that environmental factors like heavy rainfall or flooding are linked to outbreaks in humans (19), emphasizing the importance of this interplay in the disease’s epidemiology. Monitoring environmental and *in vivo* biofilms provides insight into the disease’s dynamics, which is essential for timely prevention and control strategies, ultimately improving human and animal health.

In addition to its protective properties against adverse conditions, the biofilm matrix also provides a niche for evasion from the immune system, enabling the pathogen to persist in the host and impairing treatment (39) The challenge of diagnosing and treating biofilm-related infections highlights their relevance to public health. In human medicine, biofilm-related infections are associated with higher mortality and morbidity, such as indwelling catheter infections (40), cystic fibrosis (41), endocarditis (42), and dental caries (43). In veterinary medicine, biofilms affect both production and companion animals in several diseases such as bovine mastitis, equine wounds, urinary tract infections in dogs and cats, dermatitis, and orthopedic implants (44). Therefore, it is important to understand the mechanisms involved in biofilm formation, both in the environment and within hosts, to establish appropriate control measures and health promotion strategies.

## BIOFILMS BY *LEPTOSPIRA* SPP.

The epidemiology and pathogenesis of *Leptospira* are anchored in the colonization of host tissues and survival in the environment (45). Their ability to survive outside the host indicates the presence of regulatory systems that help them adapt to various environmental challenges (46). Davignon and colleagues (38) discovered that *L. interrogans* uses an energy conservation strategy in biofilms to withstand environmental stresses, characterized by the downregulation of genes involved in motility, energy production, and metabolism (38). Chronic infections indicate the presence of factors that prevent the immune system’s effectiveness and the action of antimicrobial agents (47). Recently, Sarma and colleagues (48) demonstrated that the leptospiral cell wall hydrolase LIC_10271 contains conservated domains that primarily regulate interactions with extracellular matrix and biofilm components, predominantly within the P1 group of pathogenic *Leptospira* (48).

The negative impact of leptospirosis on human and animal health has driven the investigation of biofilms by leptospires in different settings. Table 1 shows studies on biofilm formation *in vivo* and/or addressing autochthonous strains. Leptospires were able to form biofilms *in vitro* (5, 46, 49, 50). They were isolated from environmental biofilms in an urban community with poor sanitization, suggesting their role in the ecological maintenance of the pathogen (37). In addition, a new pathogenic species (*L. interrogans* KeTo) was isolated from a sewage system (51), highlighting the need to investigate the significance of these environmental reservoirs in the transmission of leptospirosis. Previous research has shown *Leptospira* aquatic biofilms in paddy field surface waters, submerged paddy leaves, stagnant rainwater, and sewers (52). Leptospires are capable of coaggregating with other microorganisms, such as *Azospirillum* and *S. aureus* (53, 54). These interactions may have implications for the transmission of leptospirosis by facilitating the bacteria’s survival in environmental biofilms.

**TABLE 1:** Research articles on biofilm formation by *Leptospira* spp.

Sample origin	Biofilm source	Country	*Leptospira* species	Reference
Environmental wildlife enclosure	Sewage	India	Pathogenic	51
Environmental urban community setting	Rock’s surface, polyvinyl chloride pipes, and open sewage	Brazil	Saprophytic	37
Environmental rural livestock raising setting	Sediment of freshwater marshland	Brazil	Saprophytic	17
Acutely naturally infected dogs	Polystyrene plates (*in vitro*)	Brazil	Pathogenic	7
Horses with recurrent uveitis	Vitreous humor (*in vivo*)	Germany	Pathogenic	55
Chronically naturally infected Norway rats (*Rattus norvegicus*)	Renal tubules (*in vivo*)	Brazil	Pathogenic	6
Naturally infected cattle and dogs	Abiotic surface (*in vitro*)	Brazil	Pathogenic	50
Mouse model of chronic infection	Renal tubules (*in vivo*)	Japan	Pathogenic	56
Environmental urban and rural settings	Surface and submerged paddy field water and leaf; household sewers, main sewers, and stagnant rainwater	India	Pathogenic	52
Swine isolate and experimentally infected pregnant guinea pigs	Abiotic surface (*in vitro*) and placental vessels (*in vivo*)	Argentina	Pathogenic and saprophytic	57
Laboratory maintained strains	Abiotic surface (*in vitro*)	Brazil	Pathogenic and saprophytic	5

In animal hosts, biofilms have been found in the renal tubules of *Rattus norvegicus* (6), in the vitreous humor of horses with recurrent uveitis (55), and as leptospiral cell aggregates in the renal proximal tubules of infected mice (56). Strains from cattle and dogs have demonstrated strong adhesive biofilm formation *in vitro* (50). Autochthonous samples from naturally infected dogs formed mature biofilms, *in vitro*, within just 7 days of incubation (7). The biofilm-forming ability of clinical strains is crucial for understanding their role in infection persistence and therapeutic failures (7).

Ristow and colleagues (5) investigated biofilm formation by *Leptospira* driven by the ability of both saprophytic and pathogenic species to survive in aquatic environments, which is a crucial factor for the transmission of leptospirosis (5). In addition, it was believed that chronic colonization of renal tubules in maintenance hosts occurs through the formation of cell aggregates. Therefore, the initial investigation into the structure and potential mechanisms involved in the renal colonization of reservoir hosts was of fundamental importance. The authors observed that 90% of the studied strains formed biofilms *in vitro*, with pathogenic species requiring longer periods for maturation. Structurally, the biofilms were pyramid-shaped microcolonies, multicellular, and composed of several layers, with bacteria separated by channels (5).

Other currently investigated features in *Leptospira* biofilms include the role of eDNA and the Bis-(3′−5′)-cyclic diguanosine monophosphate (c-di-GMP) (46, 58, 59). eDNA was shown to be critical in maintaining structural integrity and stability, as treatment of *L. biflexa* biofilms with DNAse resulted in a significant reduction in biofilm biomass and the collapse of the matrix network (58). In *L. interrogans,* the cohesive matrix is also composed of eDNA (46). This component is crucial in biofilms, as robust and mature structures might negatively impact antimicrobial penetration (60).

c-di-GMP is a second messenger involved in biofilm formation by saprophytic and pathogenic species (46, 59). *L. biflexa* presented a varied repertoire of c-di-GMP enzymes and effectors involved in flagella assembly and interaction with other cellular systems during biofilm formation (59). The authors broadened the investigations into pathogenic *L. interrogans*, identifying eight homolog proteins that share high similarity with those found in *L. biflexa*. In a previous study, Thibeaux and colleagues (46) showed that interference with *Leptospira* c-di-GMP compromised biofilm formation and motility in *L. interrogans*. These findings suggest that targeting functional signaling and composition molecules of biofilms could be considered a promising therapeutic strategy for treating biofilm-related leptospirosis (46).

Another protein essential for maintaining *Leptospira* biofilms is the heat shock chaperone GroEL (61). Its expression confers resistance to adverse conditions, which is vital for the survival of leptospires in hostile environments, such as contaminated waters. In addition, because of its high immunogenicity, GroEL has been proposed as a potential target for new therapeutic strategies (61).

## ANTIMICROBIAL RESISTANCE

Because of the challenges in diagnosing leptospirosis, antimicrobial treatment is often empirical, resulting in varying responses to different antimicrobial agents (62–68). In addition, there are limited randomized clinical trials, both in humans and animals, and *in vitro* results may not always translate to clinical significance, particularly in leptospirosis which lacks the definition of clinical breakpoints for antimicrobials (66, 67). We highlight that the lack of consensus on MIC interpretation could lead to mismanagement of therapeutic interventions.

The agents recommended for treatment in humans include cephalosporins, doxycycline, penicillin, and macrolides (69). Penicillin, doxycycline, and streptomycin have been the agents of choice against animal leptospirosis (23, 68). In general, human strains showed less variation in their susceptibility profile for *L. interrogans* (70–73). In animals, heterogeneity could be observed for some antimicrobials, but in general, planktonic strains show susceptibility to the recommended therapeutic agents (penicillin, amoxicillin, doxycycline, and streptomycin) and resistance to sulfamethoxazole/trimethoprim and neomycin (63, 64, 66, 67).

Since treatment should start as soon as leptospirosis is suspected to reduce bacteremia and prevent urinary excretion (23), the association between biofilms and antimicrobial resistance highlights the importance of studying the susceptibility of leptospires in biofilm form. However, the difficulty in isolating *Leptospira* from the environment or clinical samples has made it challenging to track antimicrobial resistance trends in this zoonotic pathogen (67). Research on biofilm formation and its potential correlation to reduced susceptibility remains scarce.

In general, leptospires associated with biofilms are less susceptible to antimicrobials *in vitro* (7, 74, 75). De Carvalho and colleagues (7) observed that biofilm-forming strains exhibited substantially higher resistance to antibiotics compared to their planktonic forms. MIC90 was notably elevated, with values >1,600 µg/mL for doxycycline and ciprofloxacin (7). In addition, subinhibitory antibiotic concentrations have been shown to induce biofilm formation in *Leptospira*, emphasizing the risks of poor therapeutic management for leptospirosis treatment (75). Even in the planktonic form, susceptibility can vary between different strains, and it is recommended that therapeutic protocols match the specific infecting strain (66, 68).

## STRATEGIES FOR THE CONTROL AND PREVENTION OF *LEPTOSPIRA* SPP. BIOFILMS

The strong association between antimicrobial resistance and biofilm infections propels the search for new methods to disrupt mature biofilms and/or prevent their formation. While antibiotics remain the main treatment for clinical biofilm-associated infections, biofilms limit the penetration of these drugs (76). However, the production of new antibiotics is much slower than the dissemination of resistant bacteria (77). This highlights the need for alternative approaches to disrupt the biofilm multicellular structure, enhancing the effectiveness of conventional antimicrobial agents and host defense cells, by improving their access to protected cells within the biofilm matrix.

One major challenge in developing new therapeutic alternatives is ensuring they are non-toxic and environmentally friendly. Studies on nanoparticles and natural agents are increasingly reported (78, 79). Nanoformulations offer advantages such as delivering water-insoluble drugs, protecting against enzymatic reactions, lower toxicity, and crossing membranes that conventional drugs cannot (77). Natural compounds also have advantages, including higher bioavailability, less toxicity, potential synergistic effects with antibiotics, and lower MICs (76).

Nanoparticles have been tested *in vivo* for periodontitis treatment in rats (80) and *in vitro* as delivery systems for herbal extracts against *Streptococcus mutans* biofilms (81). They have also been used as silver nanoparticle formulations for Lebanese plant extracts to prevent and treat biofilms by Gram-positive and Gram-negative bacteria (82). Similarly, natural anti-biofilm agents like chestnut honey have shown effects on mixed biofilms at various stages of maturity (83), essential oils have been effective against *Staphylococcus aureus* and extended-spectrum beta-lactamase (ESBL)-producing *E. coli* mature biofilms (84, 85), and lupin seed extracts have prevented biofilm formation by Gram-positive bacteria (86). However, as of this review, no published research evaluated the anti-biofilm potential of natural agents or nanoparticles specifically against *Leptospira*.

Currently, one of the main challenges in leptospirosis is the early diagnosis of infection and the pathological processes associated (87). Likewise, a relevant aspect of managing biofilm-associated infections is the search for new diagnostic strategies to enable early intervention. Since biofilms are associated with lower treatment efficacy, delayed detection also delays prevention and treatment, with significant losses to human and animal health (88). Current biofilm detection techniques, however, primarily target mature structures and chronic infection stages (89).

Bacterial cell aggregates can be observed at the initial stages of biofilm development (32). For *L. interrogans*, these aggregates have been observed *in vitro* as early as the first day of incubation, with mature biofilms forming in just 7 days (7). *In vivo*, aggregates were found attached to the proximal renal tubules of infected mice, acting as protective and replicative structures (56), and in the placental tissues of guinea pigs (57). Bacterial aggregates benefit pathogenic bacteria, facilitating their persistence in hosts, participating in the transmission of the infectious agent, and increasing antimicrobial resistance (36, 90). Although the link between therapeutic failure and *Leptospira* aggregates remains unexplored, it was reported in dogs that the pathogen can persist after acute phase treatment, multiply, and establish chronic infections (91). Thus, future research should focus on the detection of cell aggregates and biofilms in naturally infected hosts at different stages of infection and their potential role in therapeutic failures and the establishment of *Leptospira* reservoirs.

## FUTURE PERSPECTIVES

Most studies on the environmental isolation of *Leptospira* and their association with biofilms focus on saprophytic species (17, 37), with few identifying pathogenic species in the environment (52, 92–94). The difficulty in isolating pathogenic species is attributed to (i) high concentrations of other bacteria in environmental samples, (ii) dilution of the pathogen in water samples, and (iii) the lack of a standard protocol for culturing pathogenic species from the environment (17).

Biofilms have been referred to as relevant ecological niches that help *Leptospira* survive in the environment (37, 52). A larger number of isolates and serovars from different geographical regions would help monitor their antimicrobial susceptibility patterns and their potential correlation with biofilm formation. These insights could clarify how biofilms affect transmission dynamics and highlight the need to adjust therapeutic approaches for humans and animals. In addition, although isolating leptospires from the environment is difficult, this approach could also provide insights into the survival of this pathogen, especially since one main route of infection is through contact with contaminated water or soil (37).

Further research is essential to explore the genetic, environmental, and host factors that influence biofilm formation and how they contribute to *Leptospira* persistence. Furthermore, most studies on leptospiral biofilms have largely focused on their role in the establishment and maintenance of chronic infections and environmental survival, particularly in saprophytic species. Since biofilm formation and leptospirosis pathogenesis are dynamic processes, investigations should include other clinical settings, other *Leptospira* species, and both accidental hosts and carriers.

## CONCLUSIONS

In this paper, an overview of the relationship between *Leptospira* and biofilms provided evidence that this complex structure must be considered in the survival, transmission, pathogenesis, and treatment of leptospirosis. Challenges such as the difficulty of isolating *Leptospira* from environmental and clinical samples, the lack of clinical breakpoints for leptospirosis treatment, and the heterogeneity in susceptibility profiles—especially in animals—indicate the need for further research on underlying factors affecting disease pathogenesis and treatment. Surveillance of biofilm-forming *Leptospira* should include water, soil, and various stages of infection. Since biofilms are associated with reduced susceptibility, one of the main challenges is conducting more *in vivo* studies to assess the efficacy and safety of new therapeutic molecules for biofilm disruption in animals and humans.
